# Introduction to advanced catalytic materials for energy and environmental applications

**DOI:** 10.1039/d6na90053d

**Published:** 2026-08-04

**Authors:** Manuel Ramos Murillo, Kalliopi Kousi, Eleonora Cali, Raúl Pérez Hernández, Quan Li

**Affiliations:** a Departamento de Física y Matemáticas, Instituto de Ingeniería y Tecnología, Universidad Autónoma de Ciudad Juárez Ave. Charro 450 Norte Ciudad Juárez Chihuahua C.P. 32310 Mexico manuel.ramos@uacj.mx; b School of Chemistry and Chemical Engineering, University of Surrey Surrey GU2 7XH UK k.kousi@surrey.ac.uk; c Department of Applied Science and Technology (DISAT) Politecnico di Torino 10129 Torino Italy eleonora.cali@polito.it; d Instituto Nacional de Investigaciones Nucleares Carretera México Toluca-La Marquesa s/n Ocoyoacac Estado de México C.P. 52750 Mexico raul.perez@inin.gob.mx; e Department of Physics, The Chinese University of Hong Kong Shatin Hong Kong Hong Kong

## Abstract

Manuel Ramos Murillo, Kalliopi Kousi, Eleonora Cali, Raúl Pérez Hernández and Quan Li introduce the *Nanoscale Advances* and *Catalysis Science and Technology* themed issue on *Advanced Catalytic Materials for Energy and Environmental Applications*.
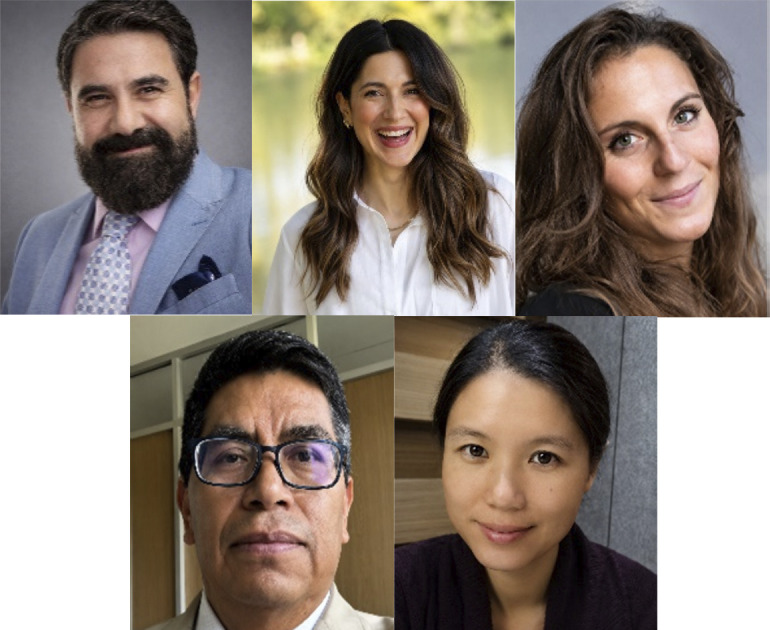

We thank all contributors to our themed collection *“Advanced Catalytic Materials for Energy and Environmental Applications”*. All the contributions included in this collection encompass a broad range of advances in heterogeneous catalysis, electrocatalysis, photocatalysis, environmental remediation, and computational catalysis. They all advance the experimental and theoretical foundations of catalytic materials science, with particular emphasis on the design, synthesis, characterization, and performance evaluation of bulk and nanoscale catalysts for sustainable energy and chemical conversion processes. Key application areas include hydrogen production through electrocatalytic pathways for fuel cell technologies, biomass valorization, energy storage systems, and organic electrosynthesis. Special attention is given to the development of photocatalytic materials, electrocatalytic materials, and single-atom catalysts.

The reported studies encompass the controlled synthesis, optimization, and scale-up of a broad range of catalytic materials, including metal–organic frameworks (MOFs), metal nanoparticles, metallocenes, ionic liquids, transition-metal carbides, phosphides, nitrides, and sulfides. Significant advances are also described in the *in situ* investigation of catalyst synthesis, activation, and pretreatment processes, providing valuable insights into structure–property relationships and catalytic functionality, including a wide spectrum of state-of-the-art characterization techniques that has been employed, including *in situ* and *operando* methodologies based on atomic force microscopy (AFM), scanning tunneling microscopy (STM), scanning and transmission electron microscopies (SEM, STEM, and TEM), X-ray photoelectron spectroscopy (XPS), synchrotron-radiation-based techniques, Raman spectroscopy, and Mössbauer spectroscopy. Complementary computational modeling, together with artificial intelligence and machine-learning approaches, has further contributed to the understanding, prediction, and optimization of catalytic materials, reaction pathways, and catalytic mechanisms.

Topics covered include electrochemical ammonia synthesis; the application of sodium borohydride for the catalytic degradation of toxic dyes; hydrogel-based materials for wastewater treatment;^1^ and the synthesis and catalytic performance of iron-based nanostructures.^2^ Significant efforts have also been devoted to energy-related catalytic processes, including dry methane reforming over Pt/CeO_2_ catalysts,^3^ selective CO_2_-to-CO conversion, and dilute methane oxidation over copper-exchanged zeolites.^4^ Several studies provide fundamental insights into catalyst design and structure–activity relationships, including investigations of operando-exsolved FeNi_3_ nanoparticles, the influence of ceria morphology on redox properties and CO oxidation activity, the catalytic behavior of supported isolated Pd complexes, and the role of Pd ensemble size in dilute and single-atom PdAu alloy catalysts.^5^ Advances in porous and functional materials are represented by research on MFI-type zeolites for ethanol dehydrogenation to acetaldehyde and highly microporous carbon supports derived from poly(vinyl chloride) (PVC) for hydrotreating applications.^6^ Environmental and photocatalytic applications are addressed through studies on the degradation of diclofenac *via* photocatalytic and radiocatalytic pathways, the development of WO_3_/Nb_2_CT_*x*_ MXene 2D–2D heterojunctions as high-performance photoanodes, and mechanistic investigations of photocatalytic processes in laser-synthesized niobium-based oxides.^7^ Complementary theoretical and computational contributions provide atomistic insights into support-induced sulfur resistance in Ni-based reforming catalysts, hydrogen adsorption phenomena, and hydrogen peroxide adsorption on cerium dioxide surfaces, thereby advancing the fundamental understanding of catalytic mechanisms and catalyst stability.^8^
